# Evaluating the Initial Experience and Clinical Impact of Prostate-Specific Membrane Antigen (PSMA) Positron Emission Tomography/Computed Tomography (PET/CT) Scans in Prostate Cancer Management: A Retrospective Study in Iraq

**DOI:** 10.7759/cureus.67814

**Published:** 2024-08-26

**Authors:** Luqman R Sulaiman

**Affiliations:** 1 Department of Medicine, Hawler Medical University College of Medicine, Erbil, IRQ

**Keywords:** diagnostics, cancer, diagnostic imaging, prostate cancer, psma pet/ct

## Abstract

Background

Prostate cancer is a significant health concern globally, especially in the Middle East, including Iraq. This study explores the adoption and impact of prostate-specific membrane antigen (PSMA) positron emission tomography/computed tomography (PET/CT) scans in Erbil, Iraq, from 2020 to 2023, marking a pivotal advancement in prostate cancer diagnostics in a region where the disease's prevalence is rising.

Materials and methods

Through a retrospective analysis at Medya Diagnostic Center in Erbil, Iraq, involving 172 patients, we assessed the feasibility, applicability, and clinical utility of PSMA PET/CT in the local population.

Results

The study highlights the modality's enhanced sensitivity and specificity in detecting prostate cancer and its metastases, with bone being the most frequent metastasis site. Despite positive outcomes, challenges such as integration into clinical practice, adherence to guidelines, and financial implications were identified. The majority of referrals came from medical oncologists, primarily for staging and response evaluation, indicating PSMA PET/CT's critical role in managing prostate cancer. The findings suggest a need for national guidelines, interdisciplinary collaboration, and educational initiatives to optimize the use of PSMA PET/CT in Iraq's healthcare setting.

Conclusions

This study contributes valuable insights into the early experiences with PSMA PET/CT, paving the way for improved prostate cancer diagnostics and management in similar contexts.

## Introduction

Prostate cancer remains a significant global health issue, with its prevalence escalating notably, especially in the Middle East and regions like Iraq [1]. In 2020, this disease accounted for approximately 1.4 million new cases and 375,000 deaths globally, reflecting a substantial increase in its incidence across various nations, including Iraq [2,3]. In Iraq, rising prostate cancer rates are further complicated by limitations in access to advanced diagnostic tools and the absence of local guidelines adapted to the region's specific healthcare needs Early diagnosis and precise staging are crucial for tailoring treatment and improving patient outcomes.

While traditional imaging modalities, including bone scans and computed tomography (CT) scans, have historically served diagnostic purposes, their limitations become evident, especially in scenarios marked by biochemical recurrence but negative conventional imaging findings [4].

In recent years, positron emission tomography (PET) with prostate-specific membrane antigen (PSMA) ligands has emerged as a promising imaging modality for prostate cancer, providing high sensitivity and specificity. Recognized for its ability to target the transmembrane protein PSMA, predominantly expressed in prostate cancer cells, this modality facilitates enhanced radiotracer uptake, thereby enabling superior lesion visualization compared to its conventional counterparts [5]. The clinical implications of PSMA PET/CT scan span various domains, including its efficacy in identifying recurrent disease subsequent to prostatectomy or radiation therapy, facilitating precise staging of high-risk prostate cancer, thereby guiding informed treatment strategies, monitoring treatment responses, and discerning disease progression patterns [6].

Despite its advantages, the integration of PSMA PET/CT into routine clinical practice in Iraq is still in its early stages. The modality was first introduced in Erbil in 2020 through the Medya Diagnostic Center, marking an important milestone in the country’s diagnostic capabilities. However, its adoption faces several anticipated challenges, including financial constraints, inadequate knowledge among healthcare providers regarding its clinical utility, and the absence of local guidelines tailored to Iraq’s unique healthcare landscape. The exclusive provision of PSMA PET/CT services by the private sector further complicates its widespread use. This situation, combined with limited insurance coverage, can lead to either overuse, imposing unnecessary financial burdens on patients, or underuse, restricting the full potential of this advanced imaging technology.

Considering the pioneering introduction of PSMA PET/CT in Iraq, our retrospective study investigates the initial experiences in Iraq between 2020 and 2023. The primary focus is on dissecting the challenges encountered during this early phase, encompassing issues related to referral practices, the appropriate use of conventional imaging prior to PSMA scan, adherence to guidelines, and other pertinent aspects. This study aspires to contribute valuable lessons that can refine the integration of PSMA in the local medical landscape and potentially serve as a reference for similar healthcare settings in Iraq.

## Materials and methods

Study design and setting

This retrospective analysis was conducted at the Medya Diagnostic Center in Erbil, Iraq, a leading facility for PSMA PET/CT imaging for prostate cancer assessment. The study period extended from January 2020 to June 2023, aiming to explore the feasibility, applicability, and impact of Ga68-PSMA PET/CT scans in staging and managing prostate cancer within the Iraqi population.

Participants

Initially, 214 cases were considered for the study. Following the application of exclusion criteria, which removed patients with other malignancies, concurrent primary cancers, or incomplete medical records, a cohort of 172 patients was finalized for evaluation. Inclusion criteria were strictly limited to patients undergoing Ga68-PSMA PET/CT scans for prostate cancer assessment.

Data collection

Data were meticulously gathered from electronic medical records, encompassing patient demographics, clinical profiles, total prostate-specific antigen (PSA) values at referral, Gleason scores, history of prior imaging modalities (CT scans, MRIs, and bone scans), clinical staging, indications for PSMA PET/CT scans (staging, follow-up, restaging, or suspected recurrence/metastases), outcomes of these scans, site of metastases if any, and specialty of the referring physician. In addition, details regarding previous PSMA PET scans and the interval between them were recorded. Table 1 elucidates these details.

**Table 1:** Summary of patient data

No.	Age	PSA level	Gleason score	CT/scan	MRI	Bone scan	Clinical stage	Reason of referral	PSMA findings	Site of mets on PSMA if any	Doctor's referral specialty
1	69	10.82	7	Not Done	Available	Not Done	T2N1M0	Staging	Negative	No Distant Metastases	Oncologist
2	56	32.3	9	Not Done	Not Done	Not Done	Not Done	Follow-up	Positive	Bone	Urologist
3	75	1.89	7	Not Done	Not Done	Not Done	Not Done	Response Evaluation	Positive	Bone and Lymph Nodes	Oncologist
4	67	7.59	9	Not Done	Available	Not Done	T2N1M0	Staging	Negative	No Distant Metastases	Urologist
5	64	74.2	9	Not Done	Not Done	Not Done	Not Done	Staging	Positive	Bone	Oncologist
6	59	14.8	7	Not Done	Available	Available	T4N2M0	Suspected Recurrence	Positive	Lymph Nodes	Radiation Oncologist
7	55	9.9	8	Not Done	Not Done	Not Done	Not Done	Staging	Negative	No Distant Metastases	Oncologist
8	59	0.25	7	Not Done	Not Done	Not Done	Not Done	Staging	Negative	No Distant Metastases	Urologist
9	72	46.95	7	Available	Available	Not Done	T4N2M1	Staging	Positive	Bone, Lymph Nodes, and Soft Tissue	Oncologist
10	80	9.1	7	Available	Available	Not Done	T2N0M0	Staging	Negative	No Distant Metastases	Oncologist
11	70	16.2	5	Available	Available	Not Done	T3N2M1	Response Evaluation	Positive	Bone	Oncologist
12	66	3.45	7	Not Done	Not Done	Not Done	Not Done	Response Evaluation	Positive	Bone	Oncologist
13	71	7	7	Available	Available	Not Done	T2N2M1	Response Evaluation	Positive	Bone	Oncologist
14	77	48.14	8	Available	Available	Not Done	T4N1M1	Staging	Positive	Bone and Lymph Nodes	Oncologist
15	55	0.307	7	Not Done	Available	Not Done	T1N0M0	Staging	Negative	No Distant Metastases	Oncologist
16	80	121.3	9	Available	Not Done	Not Done	Not Done	Suspected Recurrence	Positive	Bone, Lymph Nodes, and Soft Tissue	Oncologist
17	73	111	8	Available	Available	Not Done	T4N2M0	Staging	Positive	Bone	Oncologist
18	70	13.8	9	Not Done	Available	Not Done	Not Done	Staging	Negative	No Distant Metastases	Urologist
19	73	0.086	9	Not Done	Available	Not Done	Not Done	Response Evaluation	Negative	No Distant Metastases	Oncologist
20	70	63.64	9	Not Done	Available	Not Done	Not Done	Response Evaluation	Positive	Bone and Lymph Nodes	Oncologist
21	69	10.2	7	Not Done	Not Done	Not Done	Not Done	Suspected Recurrence	Positive	Bone	Oncologist
22	61	2.74	7	Available	Available	Available	T1N1M0	Staging	Negative	No Distant Metastases	Oncologist
23	71	8.4	6	Not Done	Not Done	Not Done	Not Done	Staging	Negative	No Distant Metastases	Oncologist
24	66	30	7	Available	Not Done	Not Done	T2N2M0	Staging	Positive	Lymph Nodes	Oncologist
25	62	3.03	7	Available	Available	Not Done	T1N1M0	Response Evaluation	Negative	No Distant Metastases	Oncologist
26	80	19.4	9	Available	Available	Not Done	T2N2M0	Response Evaluation	Negative	No Distant Metastases	Oncologist
27	55	7.19	8	Not Done	Available	Not Done	Not Done	Response Evaluation	Negative	No Distant Metastases	Oncologist
28	65	89.03	8	Available	Available	Not Done	T3N2M1	Response Evaluation	Positive	Bone, Lymph Nodes, and Soft Tissue	Oncologist
29	70	19.54	7	Not Done	Available	Not Done	Not Done	Response Evaluation	Positive	Bone and Lymph Nodes	Oncologist
30	71	6.3	7	Not Done	Available	Not Done	Not Done	Response Evaluation	Negative	No Distant Metastases	Oncologist
31	75	47.4	7	Not Done	Not Done	Not Done	Not Done	Staging	Positive	Bone, Lymph Nodes, and Soft Tissue	Oncologist
32	59	19.7	8	Available	Available	Not Done	T3N1M0	Staging	Positive	Bone	Oncologist
33	60	8.84	7	Available	Available	Not Done	T1N0M0	Staging	Negative	No Distant Metastases	Urologist
34	76	22	8	Not Done	Not Done	Not Done	Not Done	Staging	Positive	Bone and Lymph Nodes	Oncologist
35	68	0.35	6	Available	Not Done	Not Done	Not Done	Follow up	Negative	No Distant Metastases	Radiation Oncologist
36	54	19.03	8	Not Done	Available	Not Done	Not Done	Staging	Positive	Bone	Urologist
37	72	18.9	8	Available	Available	Not Done	T3N2M1	Follow up	Positive	Bone and Lymph Nodes	Oncologist
38	71	1.31	7	Not Done	Available	Not Done	Not Done	Suspected Recurrence	Negative	No Distant Metastases	Oncologist
39	69	10.42	7	Not Done	Not Done	Not Done	Not Done	Staging	Negative	No Distant Metastases	Oncologist
40	69	0.449	9	Not Done	Not Done	Not Done	Not Done	Follow up	Negative	No Distant Metastases	Oncologist
41	71	6.3	8	Not Done	Not Done	Not Done	Not Done	Suspected Recurrence	Positive	Bone	Oncologist
42	78	8.5	9	Not Done	Available	Not Done	Not Done	Suspected Recurrence	Positive	Bone	Oncologist
43	58	22.7	8	Not Done	Available	Not Done	Not Done	Staging	Positive	Bone and Lymph Nodes	Oncologist
44	58	13.6	7	Not Done	Not Done	Not Done	Not Done	Staging	Negative	No Distant Metastases	Oncologist
45	66	11.3	6	Not Done	Not Done	Not Done	Not Done	Staging	Negative	No Distant Metastases	Oncologist
46	66	231	9	Not Done	Not Done	Not Done	Not Done	Staging	Positive	Bone, Lymph Nodes, and Soft Tissue	Oncologist
47	66	49	9	Not Done	Available	Not Done	Not Done	Suspected Recurrence	Positive	Bone, Lymph Nodes, and Soft Tissue	Oncologist
48	76	9.95	6	Not Done	Not Done	Not Done	Not Done	Suspected Recurrence	Positive	Bone	Oncologist
49	57	0.11	6	Not Done	Not Done	Not Done	Not Done	Suspected Recurrence	Negative	No Distant Metastases	Radiation Oncologist
50	87	16.3	8	Not Done	Not Done	Not Done	Not Done	Staging	Positive	Bone	Oncologist
51	88	14.8	7	Not Done	Not Done	Not Done	Not Done	Staging	Positive	Bone	Oncologist
52	65	18	9	Not Done	Not Done	Not Done	Not Done	Response Evaluation	Positive	Bone and Lymph Nodes	Oncologist
53	62	16.7	7	Available	Available	Not Done	T2N0M0	Staging	Positive	Bone	Oncologist
54	76	9.5	10	Not Done	Available	Not Done	Not Done	Suspected Recurrence	Positive	Bone and Lymph Nodes	Oncologist
55	66	7.76	9	Not Done	Not Done	Not Done	Not Done	Response Evaluation	Positive	Bone	Oncologist
56	79	41.6	8	Not Done	Available	Not Done	Not Done	Staging	Positive	Bone and Lymph Nodes	Oncologist
57	68	18	5	Not Done	Available	Not Done	Not Done	Response Evaluation	Positive	Bone	Oncologist
58	64	45	8	Not Done	Not Done	Not Done	Not Done	Response Evaluation	Positive	Bone, Lymph Nodes, and Soft Tissue	Oncologist
59	58	55	9	Available	Available	Not Done	T3N1M0	Follow up	Positive	Bone and Lymph Nodes	Oncologist
60	76	8.6	7	Not Done	Not Done	Not Done	Not Done	Staging	Negative	No Distant Metastases	Oncologist
61	75	8.87	6	Available	Available	Not Done	T3N0M0	Staging	Negative	No Distant Metastases	Oncologist
62	57	51.76	9	Not Done	Not Done	Not Done	Not Done	Staging	Positive	Bone and Lymph Nodes	Oncologist
63	69	44.35	7	Not Done	Not Done	Not Done	Not Done	Staging	Positive	Bone	Oncologist
64	66	10.99	7	Not Done	Not Done	Not Done	Not Done	Response Evaluation	Positive	Bone and Lymph Nodes	Radiation Oncologist
65	78	9	8	Not Done	Not Done	Not Done	Not Done	Response Evaluation	Negative	No Distant Metastases	Radiation Oncologist
66	71	7.4	7	Not Done	Not Done	Not Done	Not Done	Staging	Negative	No Distant Metastases	Oncologist
67	55	0.04	7	Not Done	Not Done	Not Done	Not Done	Follow up	Negative	No Distant Metastases	Oncologist
68	58	7.14	7	Not Done	Available	Not Done	Not Done	Follow up	Negative	No Distant Metastases	Oncologist
69	55	6.6	8	Not Done	Not Done	Not Done	Not Done	Follow up	Negative	No Distant Metastases	Oncologist
70	75	61.8	9	Not Done	Not Done	Not Done	Not Done	Suspected Recurrence	Positive	Bone and Lymph Nodes	Urologist
71	65	8.67	9	Available	Available	Not Done	T2N1M0	Response Evaluation	Positive	Bone	Oncologist
72	75	11.35	8	Not Done	Not Done	Not Done	Not Done	Staging	Negative	No Distant Metastases	Oncologist
73	58	14.52	7	Available	Available	Not Done	T3N1M0	Response Evaluation	Negative	No Distant Metastases	Oncologist
74	77	11.08	7	Not Done	Available	Not Done	Not Done	Staging	Negative	No Distant Metastases	Radiation Oncologist
75	76	26.14	7	Not Done	Available	Not Done	Not Done	Staging	Positive	Bone, Lymph Nodes, and Soft Tissue	Oncologist
76	67	9.8	8	Available	Available	Not Done	T2N1M0	Response Evaluation	Negative	No Distant Metastases	Oncologist
77	83	1.57	9	Not Done	Available	Not Done	Not Done	Staging	Negative	No Distant Metastases	Oncologist
78	77	15.7	9	Not Done	Available	Not Done	Not Done	Staging	Negative	No Distant Metastases	Oncologist
79	73	3.1	7	Not Done	Not Done	Not Done	Not Done	Staging	Negative	No Distant Metastases	Urologist
80	72	19.28	7	Not Done	Not Done	Not Done	Not Done	Staging	Positive	Bone and Lymph Nodes	Oncologist
81	60	48.85	8	Not Done	Available	Not Done	Not Done	Staging	Positive	Bone	Oncologist
82	61	50.4	7	Not Done	Not Done	Not Done	Not Done	Staging	Positive	Bone and Lymph Nodes	Oncologist
83	60	193	7	Not Done	Available	Not Done	Not Done	Staging	Positive	Bone, Lymph Nodes, and Soft Tissue	Oncologist
84	73	19.49	6	Not Done	Available	Not Done	Not Done	Staging	Negative	No Distant Metastases	Oncologist
85	77	12l9	7	Available	Not Done	Not Done	Not Done	Staging	Positive	Bone, Lymph Nodes, and Soft Tissue	Oncologist
86	77	1.89	8	Available	Not Done	Not Done	Not Done	Response Evaluation	Negative	No Distant Metastases	Oncologist
87	70	3.2	7	Not Done	Available	Not Done	Not Done	Response Evaluation	Negative	No Distant Metastases	Oncologist
88	73	30.25	7	Available	Available	Not Done	T2N2M0	Response Evaluation	Positive	Bone	Oncologist
89	57	33.2	9	Not Done	Not Done	Not Done	Not Done	Response Evaluation	Positive	Bone and Lymph Nodes	Oncologist
90	72	12.76	9	Not Done	Not Done	Not Done	Not Done	Response Evaluation	Positive	Bone	Oncologist
91	86	5.4	8	Not Done	Not Done	Not Done	Not Done	Response Evaluation	Positive	Bone	Oncologist
92	78	13.7	7	Not Done	Not Done	Not Done	Not Done	Response Evaluation	Positive	Bone	Oncologist
93	75	8.37	8	Available	Not Done	Not Done	Not Done	Staging	Negative	No Distant Metastases	Radiation Oncologist
94	69	37.63	8	Available	Not Done	Not Done	Not Done	Staging	Negative	No Distant Metastases	Oncologist
95	80	17.43	7	Available	Available	Not Done	T3N1M0	Response Evaluation	Positive	Bone and Lymph nodes	Oncologist
96	67	7.9	7	Not Done	Not Done	Not Done	Not Done	Staging	Negative	No Distant Metastases	Oncologist
97	68	15.19	9	Available	Available	Not Done	T2N12M1	Response Evaluation	Positive	Bone	Oncologist
98	70	21.8	9	Not Done	Available	Not Done	Not Done	Staging	Negative	No Distant Metastases	Oncologist
99	67	8.3	7	Available	Not Done	Not Done	T2N1M1	Response Evaluation	Positive	Bone, Lymph Nodes, and Soft Tissue	Oncologist
100	66	11.9	7	Available	Not Done	Not Done	Not Done	Response Evaluation	Negative	No Distant Metastases	Oncologist
101	70	12.9	9	Not Done	Not Done	Not Done	Not Done	Response Evaluation	Positive	Bone	Oncologist
102	68	52.3	4	Not Done	Not Done	Not Done	Not Done	Response Evaluation	Positive	Bone and Lymph Nodes	Oncologist
103	68	7.23	8	Not Done	Not Done	Not Done	Not Done	Response Evaluation	Positive	Bone	Oncologist
104	82	43.22	9	Not Done	Not Done	Not Done	Not Done	Response Evaluation	Positive	Bone and Lymph Nodes	Oncologist
105	66	19.2	9	Not Done	Available	Not Done	Not Done	Staging	Positive	Bone	Oncologist
106	82	43.17	7	Available	Available	Not Done	T2N1M1	Staging	Positive	Bone and Lymph Nodes	Oncologist
107	68	9.3	9	Available	Available	Not Done	T2N1M0	Staging	Negative	No Distant Metastases	Oncologist
108	82	15.5	7	Available	Available	Not Done	T1N1M0	Staging	Negative	No Distant Metastases	Radiation Oncologist
109	60	0.404	9	Not Done	Not Done	Not Done	Not Done	Staging	Negative	No Distant Metastases	Oncologist
110	67	67.5	7	Available	Not Done	Not Done	Not Done	Staging	Positive	Bone	Oncologist
111	68	48.5	7	Not Done	Available	Not Done	Not Done	Staging	Positive	Bone and Lymph Nodes	Oncologist
112	73	73.9	7	Not Done	Available	Not Done	Not Done	Staging	Positive	Bone, Lymph Nodes, and Soft Tissue	Oncologist
113	77	73	8	Not Done	Available	Available	T3N1M1	Response Evaluation	Positive	Bone and Lymph Nodes	Oncologist
114	53	59.72	7	Not Done	Not Done	Not Done	Not Done	Response Evaluation	Positive	Bone and Lymph Nodes	Oncologist
115	61	25.89	8	Not Done	Not Done	Not Done	Not Done	Response Evaluation	Positive	Bone and Lymph Nodes	Oncologist
116	57	17.87	9	Not Done	Not Done	Not Done	Not Done	Response Evaluation	Positive	Bone	Oncologist
117	76	23.67	7	Not Done	Available	Not Done	Not Done	Response Evaluation	Positive	Bone	Oncologist
118	71	12.7	9	Not Done	Available	Not Done	Not Done	Staging	Negative	No Distant Metastases	Oncologist
119	71	67.2	7	Not Done	Available	Not Done	Not Done	Staging	Positive	Bone and Lymph Nodes	Oncologist
120	67	647.8	8	Not Done	Not Done	Not Done	Not Done	Staging	Positive	Bone, Lymph Nodes, and Soft Tissue	Urologist
121	71	0.09	7	Not Done	Not Done	Not Done	Not Done	Response Evaluation	Negative	No Distant Metastases	Oncologist
122	66	16.8	6	Not Done	Not Done	Not Done	Not Done	Staging	Negative	No Distant Metastases	Urologist
123	68	13.6	7	Not Done	Not Done	Not Done	Not Done	Response Evaluation	Negative	No Distant Metastases	Oncologist
124	65	41.8	7	Not Done	Not Done	Not Done	Not Done	Staging	Positive	Bone	Oncologist
125	68	29.8	8	Available	Not Done	Not Done	Not Done	Staging	Positive	Bone	Oncologist
126	72	319.5	9	Not Done	Not Done	Not Done	Not Done	Staging	Positive	Bone and Lymph Nodes	Oncologist
127	66	10.93	8	Not Done	Not Done	Not Done	Not Done	Response Evaluation	Positive	Lymph Nodes	Oncologist
128	62	9.2	9	Available	Not Done	Not Done	Not Done	Response Evaluation	Positive	Lymph Nodes	Oncologist
129	67	35.36	8	Available	Available	Not Done	T3N2M1	Response Evaluation	Positive	Bone and Lymph Nodes	Oncologist
130	78	52.7	8	Not Done	Available	Not Done	Not Done	Staging	Positive	Bone	Urologist
131	66	41.23	7	Available	Available	Not Done	T3N1M0	Staging	Positive	Bone and Lymph Nodes	Oncologist
132	62	99.45	7	Available	Not Done	Not Done	Not Done	Staging	Positive	Bone and Lymph Nodes	Oncologist
133	73	22.9	7	Not Done	Available	Not Done	Not Done	Staging	Negative	No Distant Metastases	Oncologist
134	71	92	7	Not Done	Not Done	Not Done	Not Done	Staging	Positive	Lymph Nodes	Oncologist
135	67	8.6	9	Available	Not Done	Not Done	Not Done	Staging	Negative	No Distant Metastases	Oncologist
136	77	0.5	9	Not Done	Not Done	Available	Not Done	Staging	Negative	No Distant Metastases	Oncologist
137	71	55.4	6	Not Done	Not Done	Not Done	Not Done	Staging	Positive	Bone	Oncologist
138	72	33.1	7	Not Done	Not Done	Not Done	Not Done	Staging	Negative	No Distant Metastases	Oncologist
139	65	11.9	7	Not Done	Not Done	Not Done	Not Done	Staging	Negative	No Distant Metastases	Oncologist
140	55	8.46	7	Not Done	Not Done	Not Done	Not Done	Staging	Negative	No Distant Metastases	Urologist
141	75	5.8	8	Not Done	Not Done	Not Done	Not Done	Staging	Negative	No Distant Metastases	Urologist
142	72	32.91	7	Not Done	Not Done	Not Done	Not Done	Staging	Positive	Bone	Oncologist
143	77	92.2	7	Not Done	Not Done	Not Done	Not Done	Staging	Positive	Bone and Lymph Nodes	Urologist
144	59	8.5	7	Available	Available	Not Done	T1N1M0	Staging	Negative	No Distant Metastases	Urologist
145	70	29.1	9	Not Done	Available	Not Done	Not Done	Staging	Positive	Bone and Lymph Nodes	Urologist
146	70	54.8	8	Not Done	Not Done	Not Done	Not Done	Staging	Positive	Bone and Lymph Nodes	Oncologist
147	56	5.8	9	Not Done	Not Done	Not Done	Not Done	Response Evaluation	Negative	No Distant Metastases	Radiation Oncologist
148	68	6.4	7	Not Done	Not Done	Not Done	Not Done	Response Evaluation	Positive	Bone	Oncologist
149	63	9.54	8	Not Done	Available	Not Done	Not Done	Response Evaluation	Positive	Bone and Lymph Nodes	Oncologist
150	80	0.09	7	Not Done	Not Done	Not Done	Not Done	Staging	Negative	No Distant Metastases	Oncologist
151	81	17.89	6	Available	Available	Not Done	T2N1M1	Response Evaluation	Positive	Bone, Lymph Nodes, and Soft Tissue	Oncologist
152	71	31.6	7	Available	Available	Available	T2N2M0	Staging	Positive	Lymph Nodes	Oncologist
153	65	15.37	7	Available	Not Done	Not Done	Not Done	Staging	Negative	No Distant Metastases	Oncologist
154	82	18.21	7	Not Done	Available	Available	T4N1M1	Response Evaluation	Positive	Bone and Lymph Nodes	Oncologist
155	62	21.5	7	Not Done	Not Done	Not Done	Not Done	Staging	Negative	No Distant Metastases	Oncologist
156	72	20.13	7	Not Done	Not Done	Not Done	Not Done	Staging	Positive	Bone	Oncologist
157	60	15.6	7	Not Done	Not Done	Not Done	Not Done	Staging	Positive	Lymph Nodes	Urologist
158	71	0.98	8	Not Done	Not Done	Not Done	Not Done	Response Evaluation	Negative	No Distant Metastases	Oncologist
159	77	29.23	7	Not Done	Not Done	Not Done	Not Done	Response Evaluation	Positive	Bone and Lymph Nodes	Oncologist
160	75	14.32	8	Available	Available	Not Done	T3N2M1	Response Evaluation	Positive	Bone	Oncologist
161	69	7.5	7	Not Done	Not Done	Not Done	Not Done	Staging	Negative	No Distant Metastases	Radiation Oncologist
162	73	17.29	9	Available	Not Done	Not Done	Not Done	Staging	Negative	No Distant Metastases	Oncologist
163	62	10.29	9	Not Done	Not Done	Not Done	Not Done	Staging	Negative	No Distant Metastases	Oncologist
164	75	15.2	8	Not Done	Available	Not Done	Not Done	Staging	Positive	Bone	Oncologist
165	76	24.92	7	Not Done	Not Done	Not Done	Not Done	Staging	Positive	Bone and Lymph Nodes	Oncologist
166	57	33.7	8	Not Done	Not Done	Not Done	Not Done	Staging	Positive	Lymph Nodes	Oncologist
167	61	9.05	8	Not Done	Not Done	Not Done	Not Done	Response Evaluation	Positive	Bone	Oncologist
168	68	11.7	7	Not Done	Available	Not Done	Not Done	Staging	Negative	No Distant Metastases	Urologist
169	69	9.4	7	Not Done	Available	Not Done	Not Done	Staging	Negative	No Distant Metastases	Oncologist
170	61	0.07	9	Not Done	Not Done	Not Done	Not Done	Response Evaluation	Negative	No Distant Metastases	Oncologist
171	61	18.46	7	Not Done	Not Done	Available	Not Done	Response Evaluation	Positive	Bone and Lymph Nodes	Oncologist
172	70	45.01	7	Available	Not Done	Not Done	Not Done	Staging	Positive	Bone, Lymph Nodes, and Soft Tissue	Oncologist

Imaging protocol and interpretation

A standardized protocol for Ga68-PSMA PET/CT imaging was consistently adhered to, ensuring uniformity and reliability in the imaging process. Experienced nuclear medicine professionals at the Medya Diagnostic Center interpreted the imaging results.

Statistical analysis

Data were analyzed using comprehensive descriptive statistics, including means, medians, and ranges, to summarize patient demographics, PSA levels, Gleason scores, and other key clinical measures. To investigate the associations between PSMA PET/CT findings and other parameters, various statistical tests were employed. Analyses were conducted using SPSS version 22 (IBM Corp., Armonk, NY, USA), with a significance level set at p < 0.05. 

Ethical considerations

The study protocol was approved by the Research Ethics Committee of the College of Medicine at Hawler Medical University under approval number 7-23. All procedures that contributed to this work were in accordance with the ethical standards of national and institutional human experimentation committees and the Declaration of Helsinki.

## Results

Demographic and clinical characteristics

A total of 172 cases were included in this retrospective analysis conducted at Medya Diagnostic Center, Erbil, Iraq. The study spanned from January 2020 to June 2023. The comprehensive demographic and clinical characteristics of the patients are detailed in Table 1.

The mean age of the participants was 68.8 years, ranging from 53 to 88 years, underscoring the predominance of prostate cancer in the elderly demographic. The diversity in age highlights the broad applicability of PSMA PET/CT scanning across different stages of life among the Iraqi population.

Gleason score distribution and total PSA level

**Figure 1:**
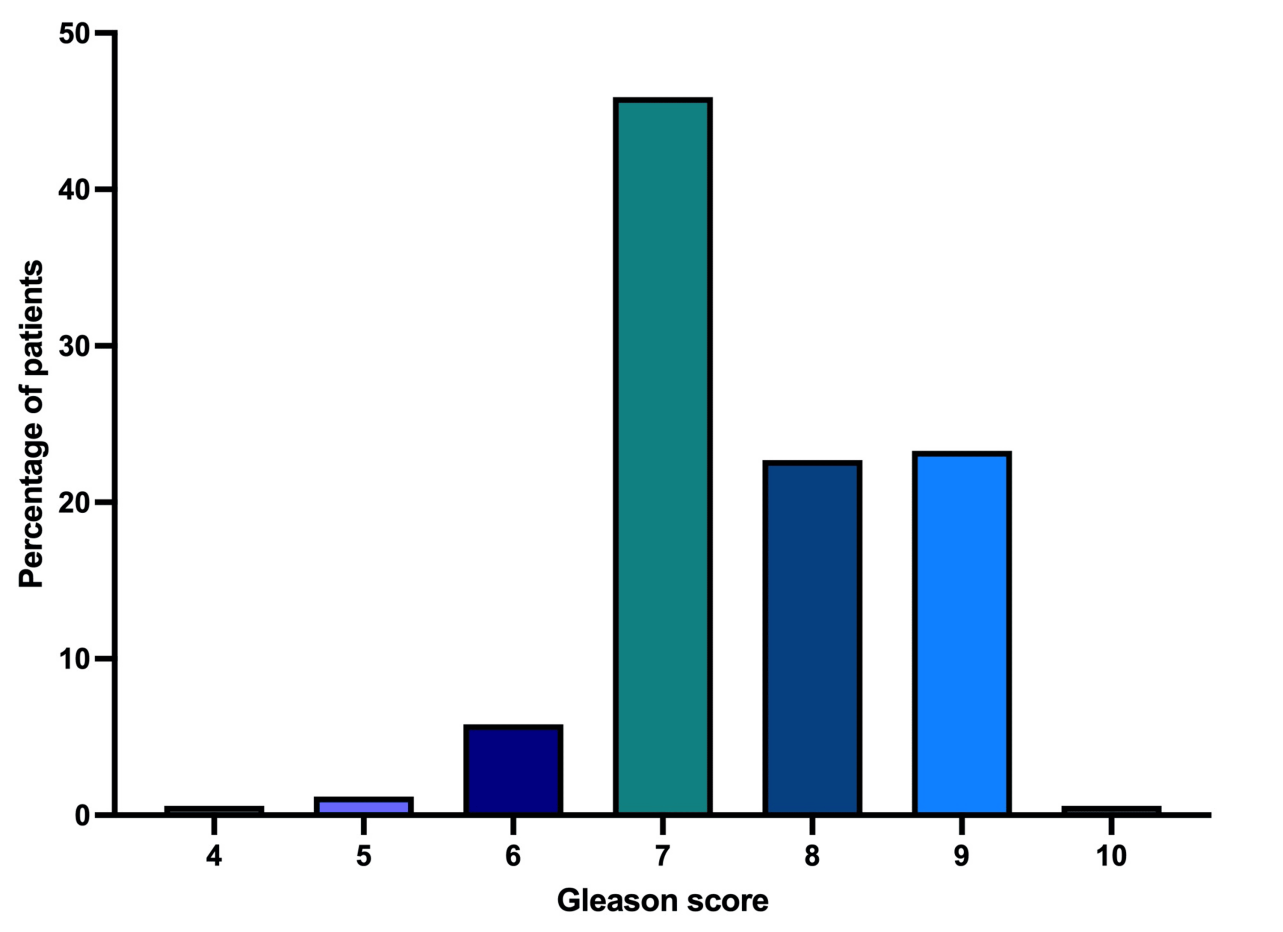
Percentage distribution of Gleason scores among the prostate cancer patients The most prevalent Gleason score was 7 (45.9%), followed by 9 (23.3%), 8 (22.7%), 6 (5.8%), 5 (1.2%), and 4 and 10 (0.6%).

The Gleason scores, a determinant of prostate cancer grade, varied from 4 to 10, with the most common score being 7 (Figure 1). This indicates a moderate differentiation of prostate cancer cells within our cohort, suggesting a prevalent intermediate risk in our patient population. The PSA levels in the cohort showed significant variability. The average PSA level was approximately 30.80 ng/mL, and the levels ranged from 0.04 to 647.80 ng/mL.

Prior imaging modalities

**Figure 2:**
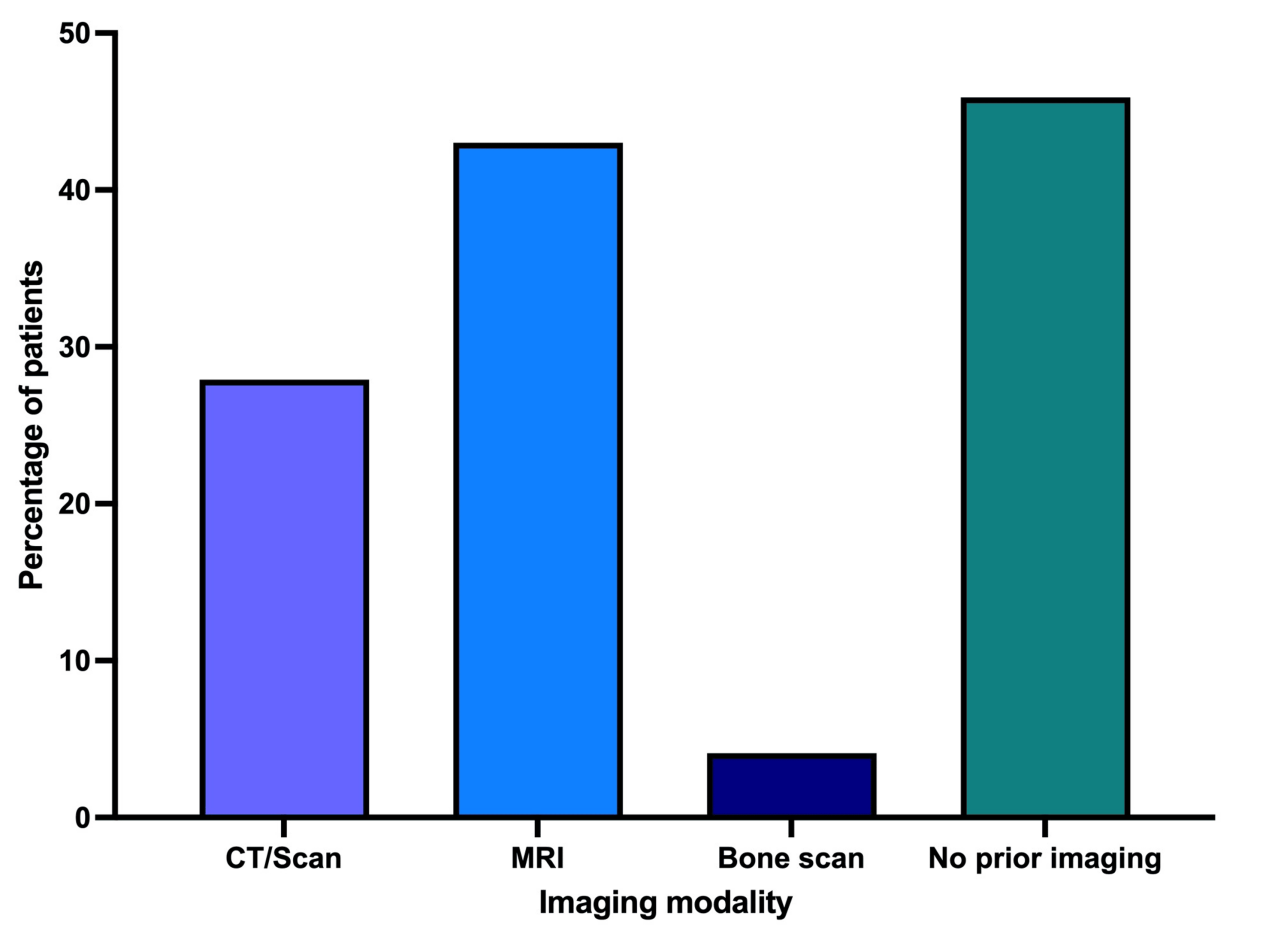
Percentage distribution of prior imaging modalities among the 172 patients Before PSMA PET/CT scans, 43.1% had MRI, 27.9% had CT, and 4.1% had bone scans.

Before undergoing PSMA PET/CT scans, a portion of the cohort had previous imaging studies: 43.1% had MRI, 27.9% had CT scans, and 4.1% underwent bone scans (Figure 2). Notably, 45.9% of the patients did not have any of these scans prior to their PSMA PET/CT, pointing to a significant reliance on PSMA PET/CT scanning for initial diagnostic assessment or staging in nearly half of the cases.

PSMA PET/CT scan outcomes

**Figure 3:**
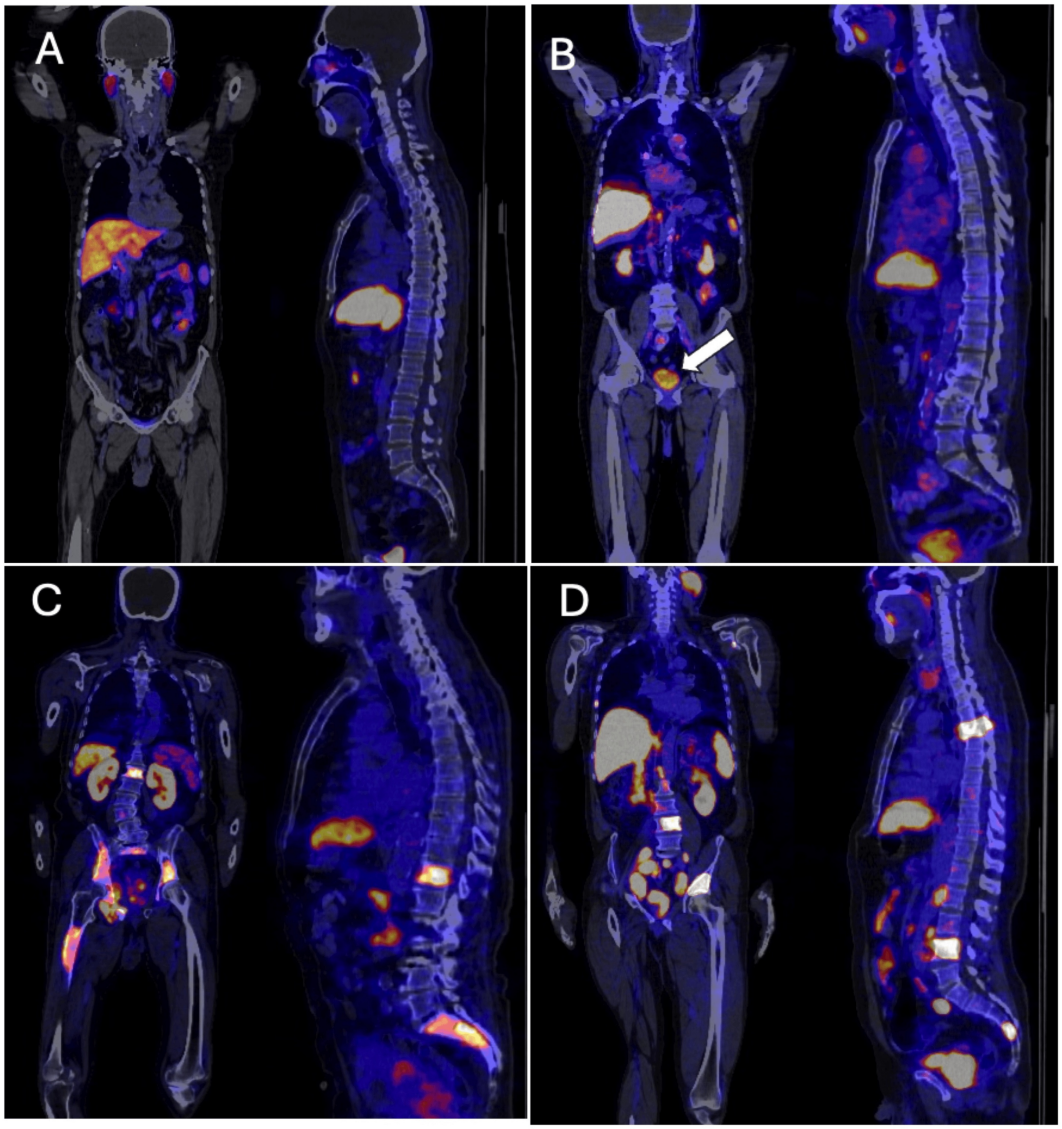
Representative PSMA PET/CT scan sample images from the cohort A. Negative PSMA PET/CT scan, indicating no detectable prostate cancer activity. B. Positive PSMA PET/CT scan confined to the prostate gland, showing localized cancer without evidence of metastasis. C. PSMA PET/CT scan revealing bone-only metastases, demonstrating PSMA uptake within the osseous structures, and DPSMA PET/CT scan displaying both bone metastases and lymph node involvement at multiple levels, indicating advanced metastatic disease with dissemination to the skeletal and lymphatic systems.

The study revealed positive PSMA scans in 59.30% (N = 102) of the patients, while 40.70% (N = 70) had negative scans. This significant rate of positive findings emphasizes the utility of PSMA PET/CT in detecting prostate cancer presence and extent, as shown in Figure 3.

Site of metastases

**Figure 4:**
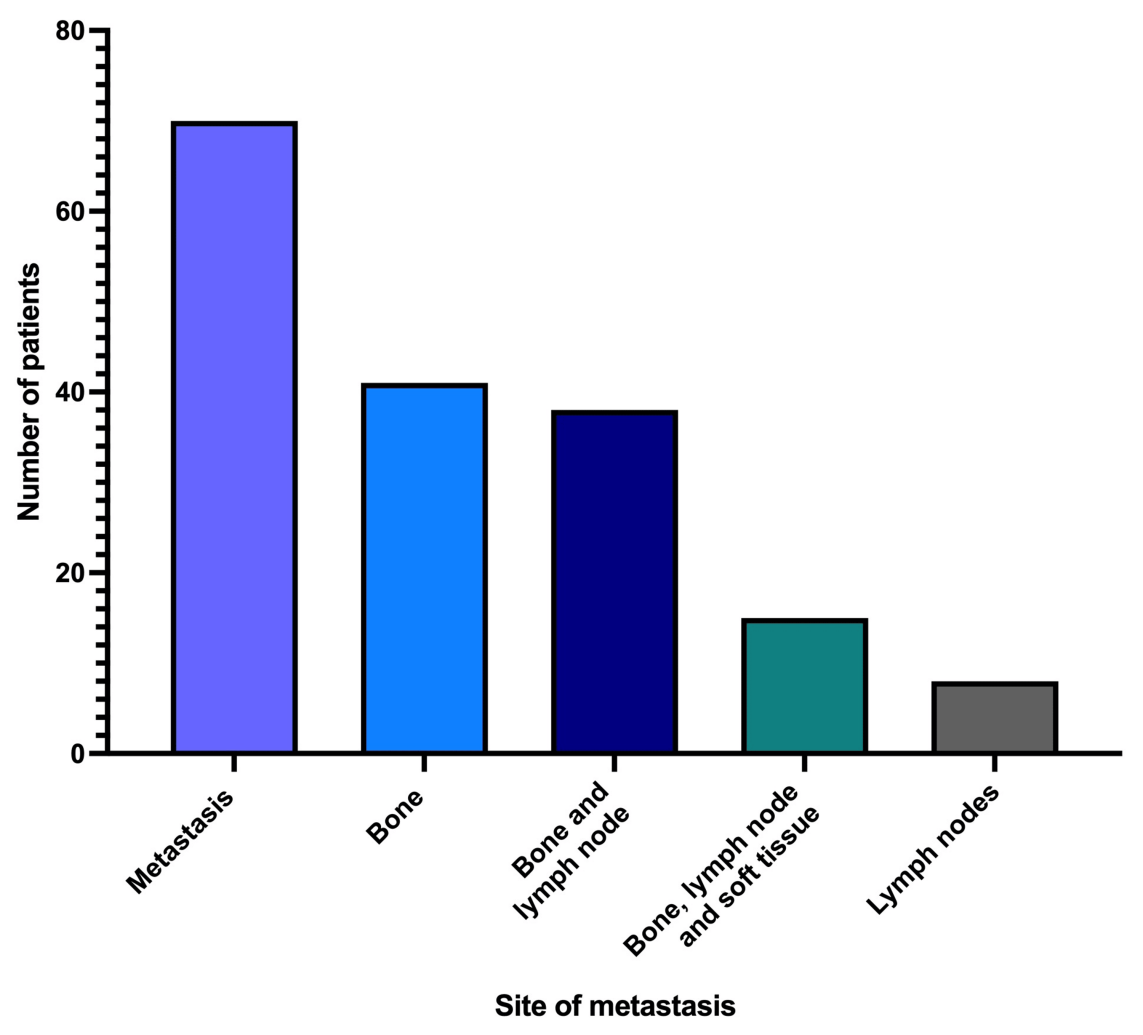
Patient distribution by the metastasis site detected in the PSMA PET/CT scans. No distance metastasis (N = 70); bone (N = 41); bone and lymph node (N = 38); bone, lymph node, and soft tissues (N = 15); and lymph nodes (N = 8). PSMA PET/CT: prostate-specific membrane antigen positron emission tomography/computed tomography

The analysis of metastatic sites in patients with positive PSMA findings revealed that the most common site was the bone (41 cases), followed by combined bone and lymph node involvement (38 cases), as shown in Figure 4. Notably, a subset of patients (15 cases) presented with metastases across all three sites (bone, lymph nodes, and soft tissue), underscoring the comprehensive diagnostic capability of PSMA PET/CT in identifying multifocal metastatic disease. In total, 70 patients (35%) had no metastases. The commonest site of metastases was bone detected in 41 cases, accounting for 20.5% of the total.

Clinical staging

Clinical staging was performed in 39 patients, with T2N1M0 being the most common stage observed, indicating localized cancer with minimal nodal involvement. However, the majority of our cohort (133 patients) lacked available clinical staging prior to the PSMA scan, primarily due to the absence of previous imaging studies, highlighting a gap in pre-PSMA diagnostic processes.

Referral patterns

**Figure 5:**
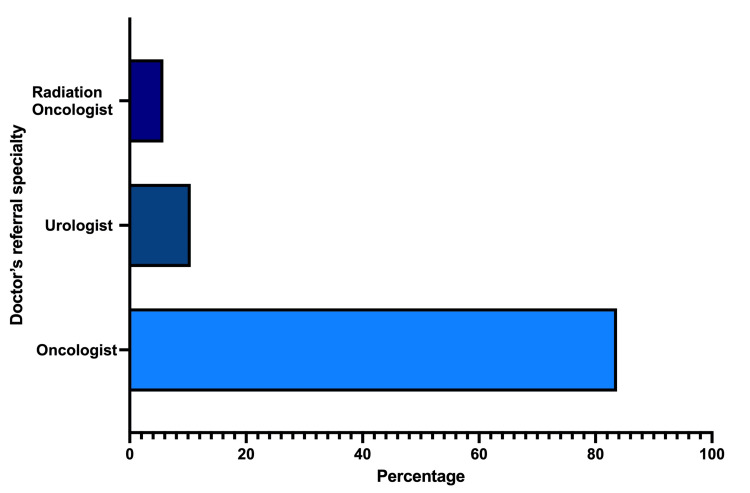
Distribution of referral specialties (%) for PSMA PET/CT scans. Oncologists referred (83.72%) for the PSMA PET/CT scans, followed by urologists (10.47%) and radiation oncologists (5.81%). PSMA PET/CT: prostate-specific membrane antigen positron emission tomography/computed tomography

The majority of referrals for PSMA PET/CT scans came from medical oncologists (N = 144, 83.72%), followed by urologists (N = 18, 10.47%) and radiation oncologists (N = 10, 5.81%), reflecting the critical role of medical oncologists in the management and referral process for prostate cancer patients in this setting (Figure 5).

Reasons for referral

**Figure 6:**
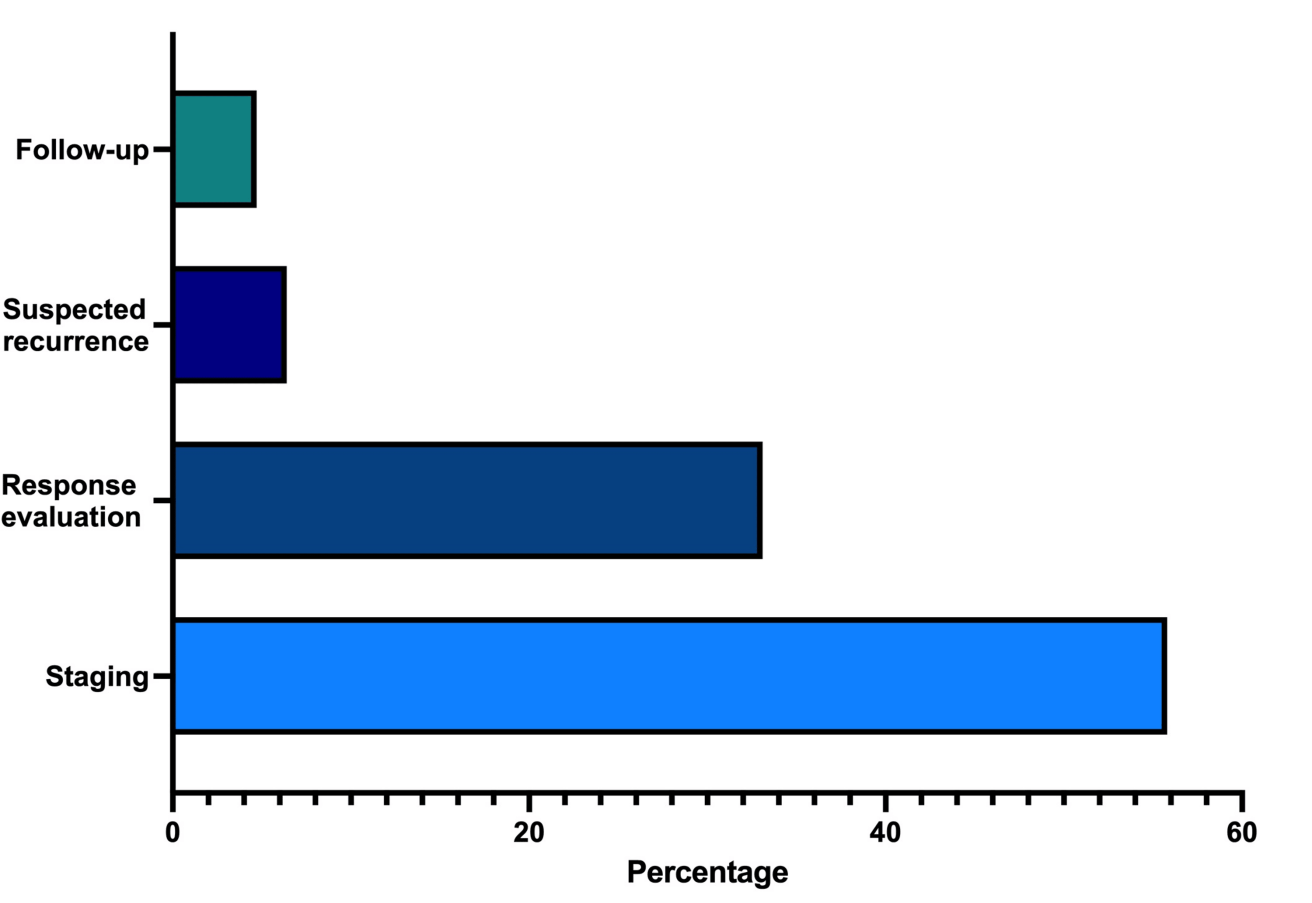
Reasons for referral. The majority of PSMA PET/CT scan referrals were for staging (55.81%), response evaluation (33.14%), suspected recurrence (6.40%), and follow-up (4.65%). PSMA PET/CT: prostate-specific membrane antigen positron emission tomography/computed tomography

Referral for PSMA PET/CT scans was primarily for staging (N = 96, 55.81%), response evaluation (N = 57, 33.14%), suspected recurrence (N = 11, 6.40%), and follow-up (N = 8, 4.65%) (Figure 6). This distribution underscores the integral role of PSMA PET/CT scans in various stages of prostate cancer management, from initial staging to monitoring treatment response and detecting recurrence.

Correlation analysis

The moderate positive correlation (r = 0.297) between PSA levels and positive PSMA findings suggests that higher PSA may be somewhat predictive of positive scans. Conversely, very weak (r = 0.056) and weak (r = 0.091) positive correlations between Gleason scores and PSA/PSMA findings indicate minimal association between these factors and positive scan outcomes.

## Discussion

The introduction of PSMA PET/CT scans at Medya Diagnostic Center in Erbil represents a significant advancement in prostate cancer diagnostics in Iraq. This innovation is particularly noteworthy given the increasing prevalence of prostate cancer in the Middle East, including Iraq, where it has become a growing health concern [7]. This study provides a comprehensive exploration of the adoption and impact of PSMA PET/CT in prostate cancer management in Erbil, Iraq, from 2020 to 2023. Our analysis highlights critical insights into the challenges and dynamics involved in integrating this advanced imaging modality within a developing healthcare infrastructure. This research not only sheds light on the initial experiences with PSMA PET/CT in the region but also underscores the broader implications of implementing such innovative technologies in areas where their application is relatively new.

The patient cohort in our study had an average age of approximately 69 years, aligning with the global trend of prostate cancer predominantly affecting older men, as observed in broader research [8]. This age profile emphasizes the critical nature of prostate cancer as a concern in aging populations. Our findings reveal substantial variability in PSA levels, highlighting the challenges in diagnosing prostate cancer based solely on these levels, which can reflect a diverse range of clinical presentations. Moreover, the commonest Gleason score of 7 in our study points to a predominance of moderately aggressive prostate cancer, similar to patterns seen in other developing regions [9]. This distribution of Gleason scores, along with the wide range of PSA levels, emphasizes the complexity involved in treatment planning and the limitations of relying exclusively on PSA levels for diagnostic and therapeutic decisions, corroborating recent academic findings [10].

The limitations of traditional imaging modalities like bone scans and CT scans, particularly in cases of biochemical recurrence with negative findings, underscore the necessity for more sensitive diagnostic tools [11]. PSMA PET/CT, with its high sensitivity and specificity, emerges as a superior alternative, especially in detecting recurrent disease post-prostatectomy or radiation therapy [12,13]. In our study, the analysis of prior imaging modalities reveals a notable trend in the diagnostic pathway for prostate cancer patients. Specifically, a significant proportion of patients were directly referred for PSMA PET/CT, bypassing more traditional, and often less expensive, diagnostic methods. This finding is critical as it suggests the potential overuse of an expensive imaging modality in scenarios where conventional methods could provide adequate diagnostic information. In a healthcare setting like Iraq, where resources are limited, a more judicious use of imaging modalities is necessary to balance the benefits of advanced techniques like PSMA PET/CT with the practicality and affordability of conventional methods like MRI, CT, and bone scans.

Therefore, while the adoption of PSMA PET/CT aligns with global advancements in prostate cancer diagnostics, it also underscores the importance of a more strategic and context-specific application of imaging resources. This would entail a careful evaluation of each patient's case to determine the most appropriate and cost-effective diagnostic pathway, ensuring optimal use of limited healthcare resources and reducing unnecessary financial burdens on patients.

The results of the PSMA PET/CT scans in our study revealed a significant proportion of positive scans, highlighting the sensitivity of PSMA PET/CT in detecting prostate cancer. However, the substantial proportion of negative scans also suggests the complexity and variability of prostate cancer presentations, underscoring the need for careful patient selection and consideration before opting for this advanced diagnostic tool.

The most common reasons for referral in our study were staging and response evaluation. This emphasizes the critical role of PSMA PET/CT in initial treatment planning and assessing treatment effectiveness, essential aspects of personalized cancer care. The utilization patterns of PSMA PET/CT in our study reflect a multifaceted approach to prostate cancer management, catering to various stages of the disease [14,15]. The multi-specialty involvement in PSMA utilization observed in our study aligns with international best practices [5]. Oncologists, urologists, and radiation oncologists all actively utilize PSMA PET/CT for staging, response evaluation, and potential treatment guidance. This interdisciplinary approach, encouraged by EAU guidelines, is crucial for maximizing the modality's benefits and tailoring patient care throughout the disease course [16]. These findings suggest that PSMA PET/CT is not only pivotal in the initial diagnosis and staging but also plays a significant role in the ongoing assessment and monitoring of prostate cancer, facilitating timely and informed therapeutic interventions.

The distribution of metastases as identified by PSMA PET/CT scans in our study provides crucial insights into the patterns of prostate cancer spread. The most common site of metastasis was bone, aligning with established knowledge that prostate cancer frequently metastasizes to bone [17]. In addition, a considerable number of patients presented with metastases in both bone and lymph nodes, indicating a more advanced stage of the disease [18]. The occurrence of metastases involving bone, lymph nodes, and soft tissue further illustrates the heterogeneity of metastatic spread in prostate cancer [19]. These findings collectively illustrate the varied patterns of prostate cancer metastasis and the pivotal role of PSMA PET/CT in accurately identifying these sites. These data also highlight the progressive nature of prostate cancer and the necessity for early and accurate detection to optimize treatment outcomes.

The most common referral reason in our study was for staging, followed by response evaluation. This pattern aligns with global trends where PSMA PET/CT is increasingly used for initial staging and assessing treatment response in prostate cancer [20]. The predominance of oncologists in referral practices reflects the central role of cancer specialists in leveraging this technology [21]. However, the varied referral reasons and specialties involved suggest the necessity for uniform guidelines and educational initiatives to optimize the use of PSMA PET/CT.

When comparing our findings with those from other studies, it is evident that the adoption of PSMA PET/CT in Iraq follows similar patterns observed in other regions, particularly in terms of its high sensitivity and specificity in detecting prostate cancer metastases. However, unlike studies conducted in more developed healthcare systems, where PSMA PET/CT is often integrated into routine clinical practice with established guidelines, our study reveals significant deviations from international guidelines, primarily due to the novelty of the technology and the lack of local protocols. For instance, the high rate of direct referrals for PSMA PET/CT without prior conventional imaging in our cohort contrasts with international practices, where PSMA PET/CT is typically reserved for cases where conventional imaging is inconclusive [16,20].

The adoption of PSMA PET/CT in Iraq faces several challenges, including limited knowledge among medical professionals, deviations from international guidelines, and financial implications. Comparative international perspectives, particularly in European countries and the USA, demonstrate more structured approaches, adherence to guidelines, and broader insurance coverage [22]. Formulating national guidelines tailored to the Iraqi healthcare landscape is essential to address these challenges. These guidelines should focus on the judicious use of PSMA PET/CT, streamline referral processes, and promote educational initiatives for healthcare professionals to enhance understanding and appropriate utilization of this modality. Furthermore, it is necessary to foster interdisciplinary collaboration among oncologists, urologists, radiologists, and nuclear medicine specialists to optimize PSMA PET/CT integration and interpretation for best clinical outcomes.

The limitations of our study, such as the retrospective design, the relatively small sample size of 172 patients, and the potential for selection bias, should be considered when interpreting the results. Furthermore, the study's duration period of 2020-2023 may not capture long-term consequences and trends. The retrospective nature limits the ability to establish causality, and the small sample size may not be representative of the broader population, reducing the generalizability of our findings. Furthermore, the financial implications of PSMA PET/CT adoption in Iraq are significant. Although a full cost-effectiveness analysis is beyond the scope of this study, it is important to note that both the overuse and underuse of this expensive imaging modality can impose additional financial burdens on the healthcare system and affect patient management. These factors highlight the need for careful consideration and strategic implementation of PSMA PET/CT in resource-limited settings like Iraq.

The implications of our findings for future research and clinical practice are substantial. Future studies should focus on larger, prospective cohorts to confirm these preliminary findings and develop comprehensive national guidelines for PSMA PET/CT usage in Iraq. Such guidelines should emphasize the judicious use of PSMA PET/CT, ensuring it is reserved for cases where it offers clear advantages over conventional imaging, and provide clear criteria for patient selection to optimize its clinical utility. In addition, interdisciplinary collaboration and educational initiatives are crucial for enhancing the understanding and appropriate utilization of PSMA PET/CT among healthcare professionals in Iraq. By addressing these areas, we can improve the integration of this advanced diagnostic tool into clinical practice and maximize its benefits for prostate cancer management in similar healthcare settings.

## Conclusions

The introduction of PSMA PET/CT at Medya Diagnostic Center marks a significant advancement in prostate cancer diagnostics in Iraq, enhancing detection accuracy and staging capabilities, particularly for metastatic disease. Despite its demonstrated utility, the integration of PSMA PET/CT faces challenges, including professional knowledge gaps, deviations from international guidelines, and financial constraints for patients. Addressing these issues necessitates the formulation of national guidelines, interdisciplinary collaboration, and educational initiatives to optimize PSMA PET/CT's use. Such efforts are crucial for maximizing the benefits of this advanced diagnostic tool, improving prostate cancer management, and potentially serving as a blueprint for similar healthcare environments.

## Appendices

**Table 2:** Summary of patient data

No.	Age	PSA Level	Gleason Score	CT/Scan	MRI	Bone Scan	cinical stage	Reasne of Referal	PSMA Findings	site of mets on PSMA if any	Dcotor's Referal Spacilaty
1	69	10.82	7	Not Done	available	Not Done	T2N1M0	Staging	Negative	No Distant Metastases	Oncologist
2	56	32.3	9	Not Done	Not Done	Not Done	Not Done	Follow up	Positive	Bone	Urologist
3	75	1.89	7	Not Done	Not Done	Not Done	Not Done	Response Evaluation	Positive	Bone and Lymphnodes	Oncologist
4	67	7.59	9	Not Done	available	Not Done	T2N1M0	Staging	Negative	No Distant Metastases	Urologist
5	64	74.2	9	Not Done	Not Done	Not Done	Not Done	Staging	Positive	Bone	Oncologist
6	59	14.8	7	Not Done	available	available	T4N2M0	Suspected Recurrence	Positive	Lymphnodes	Radiation Oncologist
7	55	9.9	8	Not Done	Not Done	Not Done	Not Done	Staging	Negative	No Distant Metastases	Oncologist
8	59	0.25	7	Not Done	Not Done	Not Done	Not Done	Staging	Negative	No Distant Metastases	Urologist
9	72	46.95	7	available	available	Not Done	T4N2M1	Staging	Positive	Bone, Lymphnodes and soft tissue	Oncologist
10	80	9.1	7	available	available	Not Done	T2N0M0	Staging	Negative	No Distant Metastases	Oncologist
11	70	16.2	5	available	available	Not Done	T3N2M1	Response Evaluation	Positive	Bone	Oncologist
12	66	3.45	7	Not Done	Not Done	Not Done	Not Done	Response Evaluation	Positive	Bone	Oncologist
13	71	7	7	available	available	Not Done	T2N2M1	Response Evaluation	Positive	Bone	Oncologist
14	77	48.14	8	available	available	Not Done	T4N1M1	Staging	Positive	Bone and Lymphnodes	Oncologist
15	55	0.307	7	Not Done	available	Not Done	T1N0M0	Staging	Negative	No Distant Metastases	Oncologist
16	80	121.3	9	available	Not Done	Not Done	Not Done	Suspected Recurrence	Positive	Bone, Lymphnodes and soft tissue	Oncologist
17	73	111	8	available	available	Not Done	T4N2M0	Staging	Positive	Bone	Oncologist
18	70	13.8	9	Not Done	available	Not Done	Not Done	Staging	Negative	No Distant Metastases	Urologist
19	73	0.086	9	Not Done	available	Not Done	Not Done	Response Evaluation	Negative	No Distant Metastases	Oncologist
20	70	63.64	9	Not Done	available	Not Done	Not Done	Response Evaluation	Positive	Bone and Lymphnodes	Oncologist
21	69	10.2	7	Not Done	Not Done	Not Done	Not Done	Suspected Recurrence	Positive	Bone	Oncologist
22	61	2.74	7	available	available	available	T1N1M0	Staging	Negative	No Distant Metastases	Oncologist
23	71	8.4	6	Not Done	Not Done	Not Done	Not Done	Staging	Negative	No Distant Metastases	Oncologist
24	66	30	7	available	Not Done	Not Done	T2N2M0	Staging	Positive	Lymphnodes	Oncologist
25	62	3.03	7	available	available	Not Done	T1N1M0	Response Evaluation	Negative	No Distant Metastases	Oncologist
26	80	19.4	9	available	available	Not Done	T2N2M0	Response Evaluation	Negative	No Distant Metastases	Oncologist
27	55	7.19	8	Not Done	available	Not Done	Not Done	Response Evaluation	Negative	No Distant Metastases	Oncologist
28	65	89.03	8	available	available	Not Done	T3N2M1	Response Evaluation	Positive	Bone, Lymphnodes and soft tissue	Oncologist
29	70	19.54	7	Not Done	available	Not Done	Not Done	Response Evaluation	Positive	Bone and Lymphnodes	Oncologist
30	71	6.3	7	Not Done	available	Not Done	Not Done	Response Evaluation	Negative	No Distant Metastases	Oncologist
31	75	47.4	7	Not Done	Not Done	Not Done	Not Done	Staging	Positive	Bone, Lymphnodes and soft tissue	Oncologist
32	59	19.7	8	available	available	Not Done	T3N1M0	Staging	Positive	Bone	Oncologist
33	60	8.84	7	available	available	Not Done	T1N0M0	Staging	Negative	No Distant Metastases	Urologist
34	76	22	8	Not Done	Not Done	Not Done	Not Done	Staging	Positive	Bone and Lymphnodes	Oncologist
35	68	0.35	6	available	Not Done	Not Done	Not Done	Follow up	Negative	No Distant Metastases	Radiation Oncologist
36	54	19.03	8	Not Done	available	Not Done	Not Done	Staging	Positive	Bone	Urologist
37	72	18.9	8	available	available	Not Done	T3N2M1	Follow up	Positive	Bone and Lymphnodes	Oncologist
38	71	1.31	7	Not Done	available	Not Done	Not Done	Suspected Recurrence	Negative	No Distant Metastases	Oncologist
39	69	10.42	7	Not Done	Not Done	Not Done	Not Done	Staging	Negative	No Distant Metastases	Oncologist
40	69	0.449	9	Not Done	Not Done	Not Done	Not Done	Follow up	Negative	No Distant Metastases	Oncologist
41	71	6.3	8	Not Done	Not Done	Not Done	Not Done	Suspected Recurrence	Positive	Bone	Oncologist
42	78	8.5	9	Not Done	available	Not Done	Not Done	Suspected Recurrence	Positive	Bone	Oncologist
43	58	22.7	8	Not Done	available	Not Done	Not Done	Staging	Positive	Bone and Lymphnodes	Oncologist
44	58	13.6	7	Not Done	Not Done	Not Done	Not Done	Staging	Negative	No Distant Metastases	Oncologist
45	66	11.3	6	Not Done	Not Done	Not Done	Not Done	Staging	Negative	No Distant Metastases	Oncologist
46	66	231	9	Not Done	Not Done	Not Done	Not Done	Staging	Positive	Bone, Lymphnodes and soft tissue	Oncologist
47	66	49	9	Not Done	available	Not Done	Not Done	Suspected Recurrence	Positive	Bone, Lymphnodes and soft tissue	Oncologist
48	76	9.95	6	Not Done	Not Done	Not Done	Not Done	Suspected Recurrence	Positive	Bone	Oncologist
49	57	0.11	6	Not Done	Not Done	Not Done	Not Done	Suspected Recurrence	Negative	No Distant Metastases	Radiation Oncologist
50	87	16.3	8	Not Done	Not Done	Not Done	Not Done	Staging	Positive	Bone	Oncologist
51	88	14.8	7	Not Done	Not Done	Not Done	Not Done	Staging	Positive	Bone	Oncologist
52	65	18	9	Not Done	Not Done	Not Done	Not Done	Response Evaluation	Positive	Bone and Lymphnodes	Oncologist
53	62	16.7	7	available	available	Not Done	T2N0M0	Staging	Positive	Bone	Oncologist
54	76	9.5	10	Not Done	available	Not Done	Not Done	Suspected Recurrence	Positive	Bone and Lymphnodes	Oncologist
55	66	7.76	9	Not Done	Not Done	Not Done	Not Done	Response Evaluation	Positive	Bone	Oncologist
56	79	41.6	8	Not Done	available	Not Done	Not Done	Staging	Positive	Bone and Lymphnodes	Oncologist
57	68	18	5	Not Done	available	Not Done	Not Done	Response Evaluation	Positive	Bone	Oncologist
58	64	45	8	Not Done	Not Done	Not Done	Not Done	Response Evaluation	Positive	Bone, Lymphnodes and soft tissue	Oncologist
59	58	55	9	available	available	Not Done	T3N1M0	Follow up	Positive	Bone and Lymphnodes	Oncologist
60	76	8.6	7	Not Done	Not Done	Not Done	Not Done	Staging	Negative	No Distant Metastases	Oncologist
61	75	8.87	6	available	available	Not Done	T3N0M0	Staging	Negative	No Distant Metastases	Oncologist
62	57	51.76	9	Not Done	Not Done	Not Done	Not Done	Staging	Positive	Bone and Lymphnodes	Oncologist
63	69	44.35	7	Not Done	Not Done	Not Done	Not Done	Staging	Positive	Bone	Oncologist
64	66	10.99	7	Not Done	Not Done	Not Done	Not Done	Response Evaluation	Positive	Bone and Lymphnodes	Radiation Oncologist
65	78	9	8	Not Done	Not Done	Not Done	Not Done	Response Evaluation	Negative	No Distant Metastases	Radiation Oncologist
66	71	7.4	7	Not Done	Not Done	Not Done	Not Done	Staging	Negative	No Distant Metastases	Oncologist
67	55	0.04	7	Not Done	Not Done	Not Done	Not Done	Follow up	Negative	No Distant Metastases	Oncologist
68	58	7.14	7	Not Done	available	Not Done	Not Done	Follow up	Negative	No Distant Metastases	Oncologist
69	55	6.6	8	Not Done	Not Done	Not Done	Not Done	Follow up	Negative	No Distant Metastases	Oncologist
70	75	61.8	9	Not Done	Not Done	Not Done	Not Done	Suspected Recurrence	Positive	Bone and Lymphnodes	Urologist
71	65	8.67	9	available	available	Not Done	T2N1M0	Response Evaluation	Positive	Bone	Oncologist
72	75	11.35	8	Not Done	Not Done	Not Done	Not Done	Staging	Negative	No Distant Metastases	Oncologist
73	58	14.52	7	available	available	Not Done	T3N1M0	Response Evaluation	Negative	No Distant Metastases	Oncologist
74	77	11.08	7	Not Done	available	Not Done	Not Done	Staging	Negative	No Distant Metastases	Radiation Oncologist
75	76	26.14	7	Not Done	available	Not Done	Not Done	Staging	Positive	Bone, Lymphnodes and soft tissue	Oncologist
76	67	9.8	8	available	available	Not Done	T2N1M0	Response Evaluation	Negative	No Distant Metastases	Oncologist
77	83	1.57	9	Not Done	available	Not Done	Not Done	Staging	Negative	No Distant Metastases	Oncologist
78	77	15.7	9	Not Done	available	Not Done	Not Done	Staging	Negative	No Distant Metastases	Oncologist
79	73	3.1	7	Not Done	Not Done	Not Done	Not Done	Staging	Negative	No Distant Metastases	Urologist
80	72	19.28	7	Not Done	Not Done	Not Done	Not Done	Staging	Positive	Bone and Lymphnodes	Oncologist
81	60	48.85	8	Not Done	available	Not Done	Not Done	Staging	Positive	Bone	Oncologist
82	61	50.4	7	Not Done	Not Done	Not Done	Not Done	Staging	Positive	Bone and Lymphnodes	Oncologist
83	60	193	7	Not Done	available	Not Done	Not Done	Staging	Positive	Bone, Lymphnodes and soft tissue	Oncologist
84	73	19.49	6	Not Done	available	Not Done	Not Done	Staging	Negative	No Distant Metastases	Oncologist
85	77	12l9	7	available	Not Done	Not Done	Not Done	Staging	Positive	Bone, Lymphnodes and soft tissue	Oncologist
86	77	1.89	8	available	Not Done	Not Done	Not Done	Response Evaluation	Negative	No Distant Metastases	Oncologist
87	70	3.2	7	Not Done	available	Not Done	Not Done	Response Evaluation	Negative	No Distant Metastases	Oncologist
88	73	30.25	7	available	available	Not Done	T2N2M0	Response Evaluation	Positive	Bone	Oncologist
89	57	33.2	9	Not Done	Not Done	Not Done	Not Done	Response Evaluation	Positive	Bone and Lymphnodes	Oncologist
90	72	12.76	9	Not Done	Not Done	Not Done	Not Done	Response Evaluation	Positive	Bone	Oncologist
91	86	5.4	8	Not Done	Not Done	Not Done	Not Done	Response Evaluation	Positive	Bone	Oncologist
92	78	13.7	7	Not Done	Not Done	Not Done	Not Done	Response Evaluation	Positive	Bone	Oncologist
93	75	8.37	8	available	Not Done	Not Done	Not Done	Staging	Negative	No Distant Metastases	Radiation Oncologist
94	69	37.63	8	available	Not Done	Not Done	Not Done	Staging	Negative	No Distant Metastases	Oncologist
95	80	17.43	7	available	available	Not Done	T3N1M0	Response Evaluation	Positive	Bone and Lymphnodes	Oncologist
96	67	7.9	7	Not Done	Not Done	Not Done	Not Done	Staging	Negative	No Distant Metastases	Oncologist
97	68	15.19	9	available	available	Not Done	T2N12M1	Response Evaluation	Positive	Bone	Oncologist
98	70	21.8	9	Not Done	available	Not Done	Not Done	Staging	Negative	No Distant Metastases	Oncologist
99	67	8.3	7	available	Not Done	Not Done	T2N1M1	Response Evaluation	Positive	Bone, Lymphnodes and soft tissue	Oncologist
100	66	11.9	7	available	Not Done	Not Done	Not Done	Response Evaluation	Negative	No Distant Metastases	Oncologist
101	70	12.9	9	Not Done	Not Done	Not Done	Not Done	Response Evaluation	Positive	Bone	Oncologist
102	68	52.3	4	Not Done	Not Done	Not Done	Not Done	Response Evaluation	Positive	Bone and Lymphnodes	Oncologist
103	68	7.23	8	Not Done	Not Done	Not Done	Not Done	Response Evaluation	Positive	Bone	Oncologist
104	82	43.22	9	Not Done	Not Done	Not Done	Not Done	Response Evaluation	Positive	Bone and Lymphnodes	Oncologist
105	66	19.2	9	Not Done	available	Not Done	Not Done	Staging	Positive	Bone	Oncologist
106	82	43.17	7	available	available	Not Done	T2N1M1	Staging	Positive	Bone and Lymphnodes	Oncologist
107	68	9.3	9	available	available	Not Done	T2N1M0	Staging	Negative	No Distant Metastases	Oncologist
108	82	15.5	7	available	available	Not Done	T1N1M0	Staging	Negative	No Distant Metastases	Radiation Oncologist
109	60	0.404	9	Not Done	Not Done	Not Done	Not Done	Staging	Negative	No Distant Metastases	Oncologist
110	67	67.5	7	available	Not Done	Not Done	Not Done	Staging	Positive	Bone	Oncologist
111	68	48.5	7	Not Done	available	Not Done	Not Done	Staging	Positive	Bone and Lymphnodes	Oncologist
112	73	73.9	7	Not Done	available	Not Done	Not Done	Staging	Positive	Bone, Lymphnodes and soft tissue	Oncologist
113	77	73	8	Not Done	available	available	T3N1M1	Response Evaluation	Positive	Bone and Lymphnodes	Oncologist
114	53	59.72	7	Not Done	Not Done	Not Done	Not Done	Response Evaluation	Positive	Bone and Lymphnodes	Oncologist
115	61	25.89	8	Not Done	Not Done	Not Done	Not Done	Response Evaluation	Positive	Bone and Lymphnodes	Oncologist
116	57	17.87	9	Not Done	Not Done	Not Done	Not Done	Response Evaluation	Positive	Bone	Oncologist
117	76	23.67	7	Not Done	available	Not Done	Not Done	Response Evaluation	Positive	Bone	Oncologist
118	71	12.7	9	Not Done	available	Not Done	Not Done	Staging	Negative	No Distant Metastases	Oncologist
119	71	67.2	7	Not Done	available	Not Done	Not Done	Staging	Positive	Bone and Lymphnodes	Oncologist
120	67	647.8	8	Not Done	Not Done	Not Done	Not Done	Staging	Positive	Bone, Lymphnodes and soft tissue	Urologist
121	71	0.09	7	Not Done	Not Done	Not Done	Not Done	Response Evaluation	Negative	No Distant Metastases	Oncologist
122	66	16.8	6	Not Done	Not Done	Not Done	Not Done	Staging	Negative	No Distant Metastases	Urologist
123	68	13.6	7	Not Done	Not Done	Not Done	Not Done	Response Evaluation	Negative	No Distant Metastases	Oncologist
124	65	41.8	7	Not Done	Not Done	Not Done	Not Done	Staging	Positive	Bone	Oncologist
125	68	29.8	8	available	Not Done	Not Done	Not Done	Staging	Positive	Bone	Oncologist
126	72	319.5	9	Not Done	Not Done	Not Done	Not Done	Staging	Positive	Bone and Lymphnodes	Oncologist
127	66	10.93	8	Not Done	Not Done	Not Done	Not Done	Response Evaluation	Positive	Lymphnodes	Oncologist
128	62	9.2	9	available	Not Done	Not Done	Not Done	Response Evaluation	Positive	Lymphnodes	Oncologist
129	67	35.36	8	available	available	Not Done	T3N2M1	Response Evaluation	Positive	Bone and Lymphnodes	Oncologist
130	78	52.7	8	Not Done	available	Not Done	Not Done	Staging	Positive	Bone	Urologist
131	66	41.23	7	available	available	Not Done	T3N1M0	Staging	Positive	Bone and Lymphnodes	Oncologist
132	62	99.45	7	available	Not Done	Not Done	Not Done	Staging	Positive	Bone and Lymphnodes	Oncologist
133	73	22.9	7	Not Done	available	Not Done	Not Done	Staging	Negative	No Distant Metastases	Oncologist
134	71	92	7	Not Done	Not Done	Not Done	Not Done	Staging	Positive	Lymphnodes	Oncologist
135	67	8.6	9	available	Not Done	Not Done	Not Done	Staging	Negative	No Distant Metastases	Oncologist
136	77	0.5	9	Not Done	Not Done	available	Not Done	Staging	Negative	No Distant Metastases	Oncologist
137	71	55.4	6	Not Done	Not Done	Not Done	Not Done	Staging	Positive	Bone	Oncologist
138	72	33.1	7	Not Done	Not Done	Not Done	Not Done	Staging	Negative	No Distant Metastases	Oncologist
139	65	11.9	7	Not Done	Not Done	Not Done	Not Done	Staging	Negative	No Distant Metastases	Oncologist
140	55	8.46	7	Not Done	Not Done	Not Done	Not Done	Staging	Negative	No Distant Metastases	Urologist
141	75	5.8	8	Not Done	Not Done	Not Done	Not Done	Staging	Negative	No Distant Metastases	Urologist
142	72	32.91	7	Not Done	Not Done	Not Done	Not Done	Staging	Positive	Bone	Oncologist
143	77	92.2	7	Not Done	Not Done	Not Done	Not Done	Staging	Positive	Bone and Lymphnodes	Urologist
144	59	8.5	7	available	available	Not Done	T1N1M0	Staging	Negative	No Distant Metastases	Urologist
145	70	29.1	9	Not Done	available	Not Done	Not Done	Staging	Positive	Bone and Lymphnodes	Urologist
146	70	54.8	8	Not Done	Not Done	Not Done	Not Done	Staging	Positive	Bone and Lymphnodes	Oncologist
147	56	5.8	9	Not Done	Not Done	Not Done	Not Done	Response Evaluation	Negative	No Distant Metastases	Radiation Oncologist
148	68	6.4	7	Not Done	Not Done	Not Done	Not Done	Response Evaluation	Positive	Bone	Oncologist
149	63	9.54	8	Not Done	available	Not Done	Not Done	Response Evaluation	Positive	Bone and Lymphnodes	Oncologist
150	80	0.09	7	Not Done	Not Done	Not Done	Not Done	Staging	Negative	No Distant Metastases	Oncologist
151	81	17.89	6	available	available	Not Done	T2N1M1	Response Evaluation	Positive	Bone, Lymphnodes and soft tissue	Oncologist
152	71	31.6	7	available	available	available	T2N2M0	Staging	Positive	Lymphnodes	Oncologist
153	65	15.37	7	available	Not Done	Not Done	Not Done	Staging	Negative	No Distant Metastases	Oncologist
154	82	18.21	7	Not Done	available	available	T4N1M1	Response Evaluation	Positive	Bone and Lymphnodes	Oncologist
155	62	21.5	7	Not Done	Not Done	Not Done	Not Done	Staging	Negative	No Distant Metastases	Oncologist
156	72	20.13	7	Not Done	Not Done	Not Done	Not Done	Staging	Positive	Bone	Oncologist
157	60	15.6	7	Not Done	Not Done	Not Done	Not Done	Staging	Positive	Lymphnodes	Urologist
158	71	0.98	8	Not Done	Not Done	Not Done	Not Done	Response Evaluation	Negative	No Distant Metastases	Oncologist
159	77	29.23	7	Not Done	Not Done	Not Done	Not Done	Response Evaluation	Positive	Bone and Lymphnodes	Oncologist
160	75	14.32	8	available	available	Not Done	T3N2M1	Response Evaluation	Positive	Bone	Oncologist
161	69	7.5	7	Not Done	Not Done	Not Done	Not Done	Staging	Negative	No Distant Metastases	Radiation Oncologist
162	73	17.29	9	available	Not Done	Not Done	Not Done	Staging	Negative	No Distant Metastases	Oncologist
163	62	10.29	9	Not Done	Not Done	Not Done	Not Done	Staging	Negative	No Distant Metastases	Oncologist
164	75	15.2	8	Not Done	available	Not Done	Not Done	Staging	Positive	Bone	Oncologist
165	76	24.92	7	Not Done	Not Done	Not Done	Not Done	Staging	Positive	Bone and Lymphnodes	Oncologist
166	57	33.7	8	Not Done	Not Done	Not Done	Not Done	Staging	Positive	Lymphnodes	Oncologist
167	61	9.05	8	Not Done	Not Done	Not Done	Not Done	Response Evaluation	Positive	Bone	Oncologist
168	68	11.7	7	Not Done	available	Not Done	Not Done	Staging	Negative	No Distant Metastases	Urologist
169	69	9.4	7	Not Done	available	Not Done	Not Done	Staging	Negative	No Distant Metastases	Oncologist
170	61	0.07	9	Not Done	Not Done	Not Done	Not Done	Response Evaluation	Negative	No Distant Metastases	Oncologist
171	61	18.46	7	Not Done	Not Done	available	Not Done	Response Evaluation	Positive	Bone and Lymphnodes	Oncologist
172	70	45.01	7	available	Not Done	Not Done	Not Done	Staging	Positive	Bone, Lymphnodes and soft tissue	Oncologist

## References

[1] Sung H, Ferlay J, Siegel RL, Laversanne M, Soerjomataram I, Jemal A, Bray F (2021). Global Cancer Statistics 2020: GLOBOCAN estimates of incidence and mortality worldwide for 36 cancers in 185 countries. CA Cancer J Clin.

[2] Hussain AM, Lafta RK (2021). Cancer trends in Iraq 2000-2016. Oman Med J.

[3] Mottet N, van den Bergh RC, Briers E (2021). EAU-EANM-ESTRO-ESUR-SIOG Guidelines on Prostate Cancer-2020 Update. Part 1: screening, diagnosis, and local treatment with curative intent. Eur Urol.

[4] Seifert R, Emmett L, Rowe SP (2023). Second version of the Prostate Cancer Molecular Imaging Standardized Evaluation Framework Including Response Evaluation for Clinical Trials (PROMISE V2). Eur Urol.

[5] Haidar M, Abi-Ghanem AS, Moukaddam H (2022). (68)Ga-PSMA PET/CT in early relapsed prostate cancer patients after radical therapy. Sci Rep.

[6] Hoffman A, Amiel GE (2023). The impact of PSMA PET/CT on modern prostate cancer management and decision making-the urological perspective. Cancers (Basel).

[7] Ali AH, Awada H, Nassereldine H, Zeineddine M, Sater ZA, El-Hajj A, Mukherji D (2022). Prostate cancer in the Arab world: bibliometric review and research priority recommendations. Arab J Urol.

[8] Bergengren O, Pekala KR, Matsoukas K (2023). 2022 update on prostate cancer epidemiology and risk factors-a systematic review. Eur Urol.

[9] Townsend NC, Ruth K, Al-Saleem T (2013). Gleason scoring at a comprehensive cancer center: what's the difference?. J Natl Compr Canc Netw.

[10] Rani E, Nibhoria S, Nagpal N (2023). Outlook of Gleason score in prostate carcinoma and correlation with PSA levels: a study in a tertiary care hospital. J Cancer Res Ther.

[11] Padhani AR, Lecouvet FE, Tunariu N (2017). Rationale for modernising imaging in advanced prostate cancer. Eur Urol Focus.

[12] Houshmand S, Lawhn-Heath C, Behr S (2023). PSMA PET imaging in the diagnosis and management of prostate cancer. Abdom Radiol (NY).

[13] Combes AD, Palma CA, Calopedos R, Wen L, Woo H, Fulham M, Leslie S (2022). PSMA PET-CT in the diagnosis and staging of prostate cancer. Diagnostics (Basel).

[14] Rasul S, Haug AR (2022). Clinical applications of PSMA PET examination in patients with prostate cancer. Cancers (Basel).

[15] Hofman M, Lawrentschuk N, Francis R (2020). Prostate-specific membrane antigen PET-CT in patients with high-risk prostate cancer before curative-intent surgery or radiotherapy (proPSMA): a prospective, randomised, multicentre study. Lancet.

[16] Gelardi F, Briganti A, Pini C, Ninatti G, Gandaglia G, Montorsi F, Chiti A (2023). European guidelines update on PSMA PET/CT for prostate cancer staging-snap back to reality. Eur J Nucl Med Mol Imaging.

[17] Ranasinghe WK, Brooks NA, Elsheshtawi MA (2020). Patterns of metastases of prostatic ductal adenocarcinoma. Cancer.

[18] Barbosa FG, Queiroz MA, Nunes RF, Viana PC, Marin JF, Cerri GG, Buchpiguel CA (2019). Revisiting prostate cancer recurrence with PSMA PET: atlas of typical and atypical patterns of spread. Radiographics.

[19] Erickson A, Hayes A, Rajakumar T (2021). A systematic review of prostate cancer heterogeneity: understanding the clonal ancestry of multifocal disease. Eur Urol Oncol.

[20] Chavoshi M, Mirshahvalad SA, Metser U, Veit-Haibach P (2022). (68)Ga-PSMA PET in prostate cancer: a systematic review and meta-analysis of the observer agreement. Eur J Nucl Med Mol Imaging.

[21] Zon RT, Goss E, Vogel VG (2009). American Society of Clinical Oncology policy statement: the role of the oncologist in cancer prevention and risk assessment. J Clin Oncol.

[22] Fendler WP, Eiber M, Beheshti M (2023). PSMA PET/CT: joint EANM procedure guideline/SNMMI procedure standard for prostate cancer imaging 2.0. Eur J Nucl Med Mol Imaging.

